# Feasibility of renal resistive index measurements performed by an intermediate and novice sonographer in a volunteer population

**DOI:** 10.1186/s13089-020-00175-6

**Published:** 2020-05-20

**Authors:** Mårten Renberg, Naima Kilhamn, Kent Lund, Daniel Hertzberg, Claire Rimes-Stigare, Max Bell

**Affiliations:** 1 Department of Perioperative Medicine and Intensive Care, Karolinska University Hospital, Solna, 171 76 Stockholm, Sweden; 2 Department of Physiology and Pharmacology, Karolinska Institutet, Stockholm, Sweden; 3 Department of Medicine, Karolinska Institutet, Solna, Stockholm, Sweden; 4 Department of Clinical Physiology, Karolinska University Hospital, Solna, Stockholm, Sweden

**Keywords:** Renal resistive index, Point-of-care ultrasound, Ultrasonography, Renal Doppler, Reproducibility of results

## Abstract

**Background:**

The Doppler-derived renal resistive index (RRI) is emerging as a promising bedside tool for assessing renal perfusion and risk of developing acute kidney injury in critically ill patients. It is not known what level of ultrasonography competence is needed to obtain reliable RRI values.

**Objective:**

The aim of this study was to evaluate the feasibility of RRI measurements by an intermediate and novice sonographer in a volunteer population.

**Methods:**

After a focused teaching session, an intermediate (resident), novice (medical student) and expert sonographer performed RRI measurements in 23 volunteers consecutively and blinded to the results of one another. Intraclass correlation coefficients and Bland–Altman plots were used to evaluate interobserver reliability, bias and precision.

**Results:**

Both non-experts were able to obtain RRI values in all volunteers. Median RRI in the population measured by the expert was 0.58 (interquartile range 0.52–0.62). The intraclass correlation coefficient was 0.96 (95% confidence interval 0.90–0.98) for the intermediate and expert, and 0.85 (95% confidence interval 0.69–0.94) for the novice and expert. In relation to the measurements of the expert, both non-experts showed negligible bias (mean difference 0.002 [95% confidence interval − 0.005 to 0.009, *p* = 0.597] between intermediate and expert, mean difference 0.002 [95% confidence interval − 0.011 to 0.015, *p* = 0.752] between novice and expert) and clinically acceptable precision (95% limits of agreement − 0.031 to 0.035 for the intermediate, 95% limits of agreement − 0.056 to 0.060 for the novice).

**Conclusions:**

RRI measurements by both an intermediate and novice sonographer in a volunteer population were reliable, accurate and precise after a brief course. RRI is easy to learn and feasible within the scope of point-of-care ultrasound.

## Background

Point-of-care ultrasound (POCUS) is ultrasonography (US) performed at the bedside by the clinician, allowing real-time interpretation of the findings [1]. In the last decades, POCUS has become an integral part of clinical decision-making in the fields of emergency medicine, critical- and perioperative care. The Doppler-derived renal resistive index (RRI) has emerged as a promising tool for assessing changes in renal perfusion in a wide range of clinical scenarios in critically ill patients [2–4]. RRI is an index derived from systolic and diastolic blood flow velocities of intrarenal arteries. Normal values are around 0.60 [5, 6] with 0.70 considered to be the upper normal threshold in adults [7]. Elevated RRI values have shown promise in early detection of acute kidney injury (AKI) in patients with shock [8–11], as well as in prognosticating intensive care unit (ICU) mortality [12]. Elevated postoperative RRI values seem to be predictive of AKI progression earlier than the conventional diagnostic criteria of oliguria and serum creatinine elevation in a broad range of major surgery [13–19]. The scope of application for RRI is expanding rapidly and the method has recently been proposed to be used in the bedside evaluation of venous congestion and fluid overload in ICU patients [20], as well as a precocious ICU monitoring tool for detecting progression and recovery from severe shock states [21].

To be clinically applicable within a POCUS protocol, RRI measurements need to be obtained by the clinician present at the bedside who may not always be an US expert. In previous studies, the examiners are described as either trained- [11, 13, 14, 17, 18, 20] or expert sonographers [8, 9, 12, 15]. In the only study comparing RRI measurements of non-expert sonographers to that of experts, interobserver reproducibility of RRI values was good after the non-experts had received a half-day course of renal Doppler [22]. These findings from centres with expertise in the RRI method have not been validated in other settings, and it is not known what specific level of US experience is needed to be able to perform RRI measurements at the bedside.

The aim of this study was to evaluate the feasibility of RRI measurements performed by two non-expert examiners, an intermediate and a novice, in a volunteer population after a focused teaching session of renal Doppler. In addition, we evaluated if there was any improvement in the agreement to an expert sonographer when the non-experts had gained practical experience from the first five examinations, hypothesizing a fast progression in the technique of obtaining RRI.

## Materials and methods

### Study population

The study involved 23 adult volunteers. The study complied with the Declaration of Helsinki and was approved by the Swedish Ethical Review Authority. Written informed consent was obtained before inclusion.

### Education of examiners

All measurements and calculations were performed by three examiners of different US experience. The intermediate examiner (MR) was a resident in anaesthesia and intensive care, using US regularly in clinical practice but without any prior experience of renal Doppler. The novice examiner (NK) was a 4th year medical student with prior experience limited to basic theory of US. The expert examiner (KL) was a specialist in clinical physiology, performing US examinations daily with more than 20 years of experience of renal Doppler and RRI measurements. The two non-experts were taught Doppler evaluation of renal perfusion on two separate occasions of 3 h each by the expert. The sessions included a basic theoretical background of renal US and supervised practical training to locate the kidneys, identify the intrarenal vessels using colour-Doppler, and measuring and calculating the RRI.

### Data collection

All examinations were performed at the Karolinska University Hospital between June and September 2019. The following variables were recorded from each volunteer: age, weight, height, heart rate, heart rhythm and resting blood pressure. Medical history and ongoing medications were recorded. Each volunteer was examined by all three examiners consecutively, the order of the examiners being random for every session. The examiners were blinded to the examinations and results of one another.

### RRI measurement and calculation

In each volunteer, the same designated ultrasound device (GE Vivid S70N, v202CH, US) with a curvilinear probe (1.5–6.0 MHz) was used. The volunteer was positioned on their side and first a complete view of the kidney was obtained. Colour-Doppler was applied to visualize the global organization of intrarenal blood vessels. Pulsed wave Doppler at the smallest possible width between 2 and 5 mm was used to measure flow velocities in an interlobular- or arcuate artery in the upper, middle and lower pole of each kidney. If possible, the examiners obtained a reading with at least three consecutive similar-looking waveforms in each of the three poles for each kidney. RRI was calculated for each of the three poles as [(peak systolic velocity − end diastolic velocity)/peak systolic velocity]. These values were used to compute a total mean RRI (RRI_total_), a mean RRI for the right kidney (RRI_dx_) and a mean RRI for the left kidney (RRI_sin_). If the examiners were unable to obtain a satisfactory measurement in one pole, the mean value was calculated using the measurements obtained. Our protocol for obtaining RRI is in line with previously described protocols [23].

### Statistical analysis

Results are described as medians with interquartile range (IQR) and minimum/maximum values (min/max) for continuous variables, or numbers and percentages for categorical variables. Interobserver reliability was assessed calculating the intraclass correlation coefficient (ICC) and their 95% confidence intervals (CI) based on an individual measurement, consistency of agreement, 2-way mixed-effects model [24]. The values were interpreted using the Ko and Li classification system [25] where < 0.5 is poor reliability, 0.50–0.74 is moderate reliability, 0.75–0.89 is good reliability, and ≥ 0.90 is excellent reliability. The mean difference in RRI measurements between non-expert examiners relative to the expert was compared using paired *t* tests. Bland–Altman plots were constructed plotting the difference of the paired measurements from respective non-expert and the expert (y-axis) against the mean of the two measurements (*x*-axis) [26]. Bias, reflecting systematic differences, was defined as the mean difference of the paired measurements [27]. Precision, reflecting random differences, was evaluated using the 95% limits of agreement (LoA) (mean difference ± 1.96 standard deviations [SD]) between paired measurements. There is no previously agreed definition of acceptable precision for RRI measurements. We considered precision to be clinically acceptable when the percentage error from a proposed normal RRI value of 0.60 was no more than ± 10%, corresponding to an LoA of ± 0.06 for the non-expert examiners in relation to the results of the expert. To evaluate any potential progression in the technique of obtaining RRI in the non-expert examiners, ICC, mean difference, and LoA were again generated after excluding the first five volunteers per examiner. Data analysis was performed using Stata version 15.1 (StataCorp, College Station, US).

## Results

### Population characteristics

Characteristics of the volunteers are described in Table 1. Two volunteers were being followed in primary care due to slightly increased serum creatinine levels. One volunteer had antihypertensive treatment. Another volunteer was found to have an asymptomatic hypermobile kidney and was referred to specialist care for follow-up.

**Table 1:** Volunteer characteristics

Number of volunteers	23
Age (years), median (IQR)	38 (31–49)
Female, *n* (%)	14 (61)
Heart rate at examination (beats/min), median (IQR)	59 (56–67)
Systolic blood pressure (mmHg), median (IQR)	117 (109–126)
Diastolic blood pressure (mmHg), median (IQR)	71 (64–83)
History of renal disease, *n* (%)	3 (13)
Elevated s-Cr levels, *n* (%)	2 (9)
Hypertension, *n* (%)	1 (4)
Antihypertensive medication, *n* (%)	1 (4)
Body mass index (kg/m^2^), median (IQR)	24 (23–26)

*IQR* interquartile range, *n* number, *s-Cr* serum creatinine

### RRI measurements

All examiners were able to obtain RRI values in all volunteers. Out of 138 possible kidney pole measurements, the intermediate was able to obtain 136 (99%), the novice 134 (97%) and the expert 138 measurements (100%). In all cases where a kidney pole measurement was missing, at least two kidney pole measurements per kidney could be recorded. Measured by the expert, the median RRI_total_ in the study population was 0.58 (IQR 0.52–0.62, min/max 0.46/0.65). All examiners measured RRI values < 0.70 in all volunteers. There was no apparent difference in RRI values obtained from the right or left kidney.

### Comparison between non-experts and expert

**Fig. 1:**
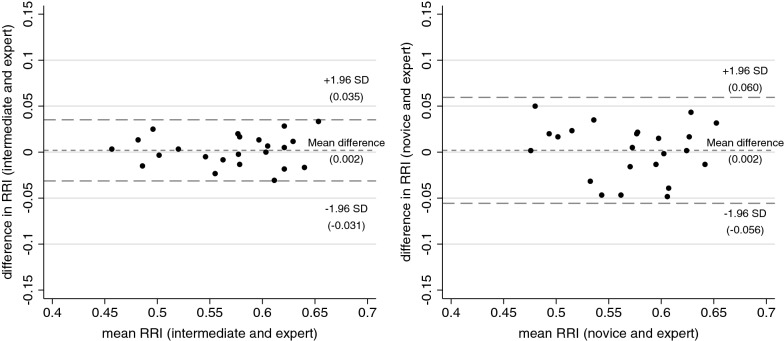
Bland Altman plots showing the comparison between renal resistive index (RRI) measurements by the intermediate and expert examiner and the novice and expert examiner. Bias is indicated by the mean difference between respective non-expert and the expert, and precision is indicated by the 95% limits of agreement represented by the mean ± 1.96 standard deviations (SD)

For RRI means (RRI_total_, RRI_dx_ and RRI_sin_) ICC, mean difference between paired measurements, and LoA for respective non-expert in relation to the expert are presented in Table 2a. Interobserver reliability for the intermediate and expert examiners was excellent (ICC ≥ 0.90) for RRI_total_ and in the range of good to excellent (ICC ≥ 0.75) for RRI_dx_ and RRI_sin_. For the novice and expert examiners, interobserver reliability was in the range of moderate to excellent (ICC ≥ 0.50) for all RRI means. There was no difference in any corresponding RRI means obtained by neither of the non-experts compared to the expert (*p* > 0.05 for all mean differences between paired measurements). Bland–Altman plots for RRI_total_ are presented in Fig. 1. For both non-experts bias was negligible (mean difference 0.002 [95% CI − 0.005 to 0.009, *p* = 0.597] between intermediate and expert, mean difference 0.002 [95% CI − 0.011 to 0.015, *p* = 0.752] between novice and expert) and precision was clinically acceptable (LoA − 0.031 to 0.035 for the intermediate, LoA − 0.056 to 0.060 for the novice).

**Table 2:** Comparison of RRI measurements between respective non-expert and expert examiners

	ICC (95% CI)	Mean difference (95% CI, p value)	LoA
(a) Total study population (*n* = 23)			
RRI_total_			
INT:EXP	0.96 (0.90–0.98)	0.002 (− 0.005 to 0.009, 0.597)	− 0.031 to 0.035
NOV:EXP	0.85 (0.69–0.94)	0.002 (− 0.011 to 0.015, 0.752)	− 0.056 to 0.060
RRI_dx_			
INT:EXP	0.94 (0.87–0.98)	0.003 (− 0.006 to 0.011, 0.508)	− 0.035 to 0.040
NOV:EXP	0.86 (0.70–0.94)	0.002 (− 0.010 to 0.015, 0.701)	− 0.054 to 0.058
RRI_sin_			
INT:EXP	0.94 (0.87–0.98)	0.001 (− 0.007 to 0.010, 0.729)	− 0.037 to 0.040
NOV:EXP	0.79 (0.57–0.91)	0.002 (− 0.014 to 0.017, 0.836)	− 0.070 to 0.073
(b) Study population after excluding first five volunteers (*n* = 18)			
RRI_total_			
INT:EXP	0.97 (0.92–0.99)	0.004 (− 0.004 to 0.012, 0.287)	− 0.026 to 0.034
NOV:EXP	0.90 (0.75–0.96)	0.008 (− 0.005 to 0.020, 0.223)	− 0.042 to 0.058

*RRI* renal resistive index, *n* number, *ICC* intraclass correlation coefficient, *CI* confidence interval, *LoA* 95% limits of agreement, *INT:EXP* comparison of intermediate and expert examiners, *NOV:EXP* comparison of novice and expert examiners

After excluding the first five volunteers, both non-experts were able to obtain 108 measurements (100%) from 108 possible kidney poles. Table 2b presents the repeated analyses for RRI_total_. ICC for both non-experts in relation to the expert increased, the novice now reaching good to excellent reliability (ICC ≥ 0.75). Bias for both non-experts was still small, and precision was slightly improved.

## Discussion

This is the first study to investigate the feasibility of RRI measurements performed by non-expert examiners on different and clearly specified prior US experience levels. In a volunteer population the intermediate examiner, a resident, showed excellent reliability compared to an expert after a brief course of renal Doppler. The novice examiner, a US-naïve medical student, showed moderate to excellent reliability compared to the same expert that increased to the range of good to excellent when the first few examinations were excluded from the analysis indicating a fast progression in the technique of obtaining RRI. The measurements by both non-experts were accurate with clinically acceptable precision. Our results suggest that RRI measurements are feasible for non-expert examiners after only a brief course.

Although several studies have shown good interobserver correlation of RRI measurements between expert sonographers [15, 28–30], only one previous study by Schnell and co-workers [22] did include non-expert examiners. In the Schnell study, residents with prior training in US for critical care patients underwent a comparable course of renal Doppler to that of our study, and then measured RRI in mechanically ventilated patients. ICC in relation to experts was 0.89 (95% CI 0.82–0.93), indicating good to excellent reliability using the Ko and Li classification system [25]. Compared to our study, this is in line with the level of interobserver correlation between the medical student and the expert, whilst the resident instead showed solely excellent reliability compared to the expert from the start. The better correlation of measurements performed by the non-experts in our study could be explained by the fact that our study population consisted of volunteers that were fairly easy to examine, and able to cooperate with breath-hold to minimize motion of the kidneys during measurements. However, measurement conditions in patients on controlled mechanical ventilation could be optimized by initiating an inspiratory- or expiratory pause during the measurement, rendering the kidneys more stationary. It can be argued that RRI values may be hardest to obtain in spontaneously breathing tachypnoeic patients, and the level of US experience and training of renal Doppler needed to obtain reliable RRI values in such a population are still to be investigated.

Only the clinical context can determine the specific demands for precision when comparing RRI values obtained by different examiners. In a study evaluating interobserver variability of RRI measurements in patients with renal allografts, an interobserver interval of − 0.035 to 0.044 between two trained sonographers was described as acceptable [31]. Another study on postoperative cardiac surgery patients deemed an LoA as wide as − 0.024 to 0.114 comparing a trained sonographer to an expert as adequate [20], though this may be questioned from a clinical standpoint. For example, in ten studies included in a meta-analysis evaluating the role of RRI to predict postoperative AKI, the mean difference of RRI between the group developing AKI and the group that did not was only 0.07 [32]. Whilst the non-expert examiners in the Schnell study [22] showed an evident lack of precision in their RRI measurements compared to experts (LoA − 0.107 to 0.105), both non-experts in our study showed far better precision. The very narrow range of the LoA for the intermediate examiner indicates a good precision that would be unlikely to affect decision-making in clinical practice. It is notable that also the novice, without prior US experience, whilst showing a wider LoA than the intermediate still had an acceptable range within ± 0.06 from the results of the expert. Previous studies have shown that US-naïve medical students were able to obtain fair image acquisition of the kidneys after focused courses [33, 34], but this is the first study to investigate and propose that also intrarenal Doppler readings by US-naïve examiners are feasible.

All RRI values obtained by all examiners in our study were within normal range, meaning neither of the non-experts measured an elevated RRI when the expert did not. There was no considerable difference in feasibility if RRI values were obtained from a mean from both kidneys, or only the right or left kidney. However, for measurements obtained from the left kidney the novice showed a worse precision with an LoA that was no longer within the clinically acceptable range. This may suggest a more difficult examination on the left side, which is in line with previous studies where RRI means sometimes were obtained only from the right kidney because it was perceived as technically easier [22, 35].

The results of our study suggest that it is possible to educate non-expert US examiners, and therefore most clinicians working with critically ill patients, the method of RRI during a brief, focused course. However, it must be stressed that interpretation of RRI measurements in these patients is complex and may still need consultation with more experienced clinicians. As POCUS is gaining interest in the management of AKI patients [36] and image acquisition of the kidneys already is part of the curriculum of basic POCUS training [37], it is not unreasonable to add the use of renal Doppler to existing POCUS training programs. Before this can happen, also the input of RRI for monitoring and therapeutic actions in various clinical contexts must be further established.

Our study has several limitations. First, the population size was small, but in line with previous RRI validation studies amongst trained sonographers [31, 38, 39]. Second, since our population consisted only of volunteers, results may not necessarily be transferred to all other hospitalized populations such as ICU patients. However, our results should be transferrable to some previously studied patient populations, for example it has been shown that preoperative RRI obtained under conditions comparable to those of our study could predict postoperative AKI [40]. Third, we only included one non-expert examiner of every experience level. These examiners were perceived as representatives for their respective level of prior US experience but were still individuals from within those groups. Caution should therefore be taken when extrapolating the results to other non-expert examiners. Finally, we did not assess intraobserver variability of RRI measurements in the examiners. Previous studies have shown the intraobserver variability to be low amongst expert- [12, 28] and trained sonographers [17, 31, 41]. The fact that there was good to excellent interobserver correlation in our study whilst examining the subjects consecutively suggests the intraobserver variability was unlikely to be of vast significance.

## Conclusions

RRI measurements performed by both an intermediate and novice sonographer in a volunteer population were reliable, accurate and precise after a brief course of renal Doppler. RRI is easy to learn with fast progression, and is feasible within the scope of POCUS.

## Data Availability

The datasets used and analysed during the current study are available from the corresponding author on reasonable request.

## Abbreviations

POCUS: Point-of-care ultrasound
US: Ultrasonography
RRI: Renal resistive index
AKI: Acute kidney injury
ICU: Intensive Care Unit
